# Toll‐like receptors in health and disease

**DOI:** 10.1002/mco2.549

**Published:** 2024-04-29

**Authors:** Kunyu Wang, Hanyao Huang, Qi Zhan, Haoran Ding, Yi Li

**Affiliations:** ^1^ Department of Head and Neck Oncology Surgery, State Key Laboratory of Oral Diseases & National Clinical Research Center for Oral Diseases West China Hospital of Stomatology Sichuan University Chengdu China; ^2^ Department of Oral and Maxillofacial Surgery, State Key Laboratory of Oral Diseases & National Clinical Research Center for Oral Diseases West China Hospital of Stomatology Sichuan University Chengdu Sichuan China

**Keywords:** cancer, clinical treatment, disease, innate immunity, toll‐like receptors

## Abstract

Toll‐like receptors (TLRs) are inflammatory triggers and belong to a family of pattern recognition receptors (PRRs) that are central to the regulation of host protective adaptive immune responses. Activation of TLRs in innate immune myeloid cells directs lymphocytes to produce the most appropriate effector responses to eliminate infection and maintain homeostasis of the body's internal environment. Inappropriate TLR stimulation can lead to the development of general autoimmune diseases as well as chronic and acute inflammation, and even cancer. Therefore, TLRs are expected to be targets for therapeutic treatment of inflammation‐related diseases, autoimmune diseases, microbial infections, and human cancers. This review summarizes the recent discoveries in the molecular and structural biology of TLRs. The role of different TLR signaling pathways in inflammatory diseases, autoimmune diseases such as diabetes, cardiovascular diseases, respiratory diseases, digestive diseases, and even cancers (oral, gastric, breast, colorectal) is highlighted and summarizes new drugs and related clinical treatments in clinical trials, providing an overview of the potential and prospects of TLRs for the treatment of TLR‐related diseases.

## Graphical abstract

Interactions of TLRs with inflammation and tumor. TLRs are expressed on a variety of cells, including tumor cells and immune cells. TLRs signaling is involved in the carcinogenesis of the tumor microenvironment. TLRs expressed on immune cells and tumor cells are subjected to activation by pathogen‐associated molecular patterns,damage‐associated molecular patterns from microorganisms, viruses, parasites, injured and necrotic cancer cells. The release of cytokines and chemokines by these activated cells is an important part of the tumor microenvironment. Moreover, cytokine‐activated infiltrating immune cells can subsequently induce additional cytokines that impair the function of antigen‐presenting cells, leading to tumor immune tolerance.

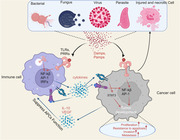

## 1 INTRODUCTION

Once thought to be a general immune response, innate immunity serves as the human being's first line of protection against microbial invasion. On the other hand, the identification of Toll‐like receptors (TLRs) brought about the first awareness that innate immunity is pathogen‐specific.^1^ The innate immune system uses germline‐encoded pattern recognition receptors (PRRs) as its first line of defense against microorganisms.^2^ Pathogen‐associated molecular patterns (PAMPs), which are chemicals specific to microbes, and damage‐associated molecular patterns (DAMPs), which are molecules derived from injured or dying cells, are recognized by TLRs, which are PRRs. Innate immune responses are induced when TLRs trigger downstream signaling pathways by generating type I interferons (IFNs), inflammation‐inducing cytokines, and other agents. These mechanisms not only set off an immediate defensive reaction from the host, but they also initiate and orchestrate an adaptive immune response specific to an antigen.^3^

TLRs are an essential part of the adaptive immune response. When stimulated, TLRs trigger downstream signaling pathways that maintain host microecological homeostasis and remove dead or mutated cells. General autoimmune diseases and both chronic and acute inflammatory conditions can result from inappropriate TLR stimulation. Moreover, an increasing body of research suggests that endogenous chemicals generated by dying cells or in specific pathogenic settings activate TLRs, which can cause or hasten the onset of autoimmune disorders and inflammation. There are several ways in which the inflammatory response can encourage the development of cancer, such as nuclear factor‐κB's (NF‐κB's) antiapoptotic action, which damages DNA oxidatively and triggers a healing response in the tissue.^4^, ^5^, ^6^, ^7^, ^8^ The pathophysiology of various malignancies has been linked to deregulation of NF‐κB, which activates through a pathway dependent on myeloid differentiation primary‐response protein 88(MyD88), causing inflammation and encouraging the conversion of precancerous cells into malignant cells.^9^

This article reviews the recent discoveries in the molecular and structural biology of TLRs; focuses on the roles of different TLR signaling pathways in inflammatory diseases, autoimmune diseases, and even cancers; and summarizes new drugs and related clinical treatments in clinical experiment, giving a general summary of TLRs’ potential and future possibilities for treating disorders linked to TLRs.

## 2 TLR AND LIGAND‐RECOGNITION MECHANISMS

TLR is a type I transmembrane protein that consists of three main structural regions. It is characterized by an Leucine‐rich repeat(LRR) in the outer domain, a membrane‐spanning structural domain, and homology domain in the cytoplasmic Toll/IL‐1R (TIR).^10^ So far, 10 functional TLRs have been identified in humans (TLR1–10), and 12 have been identified in mice (TLR1–9 and TLR11–13). Both nonimmune and immune‐related innate cells, such as fibroblasts and epithelial cells (ECs), as well as macrophages, lymphocytes, granulocytes, and dendritic cells (DCs), express them.^11^ Owing to retroviral insertion, TLR10 is not functional in mice, whereas TLR11, TLR12, and TLR13 are absent from the human DNA.

**FIGURE 1:**
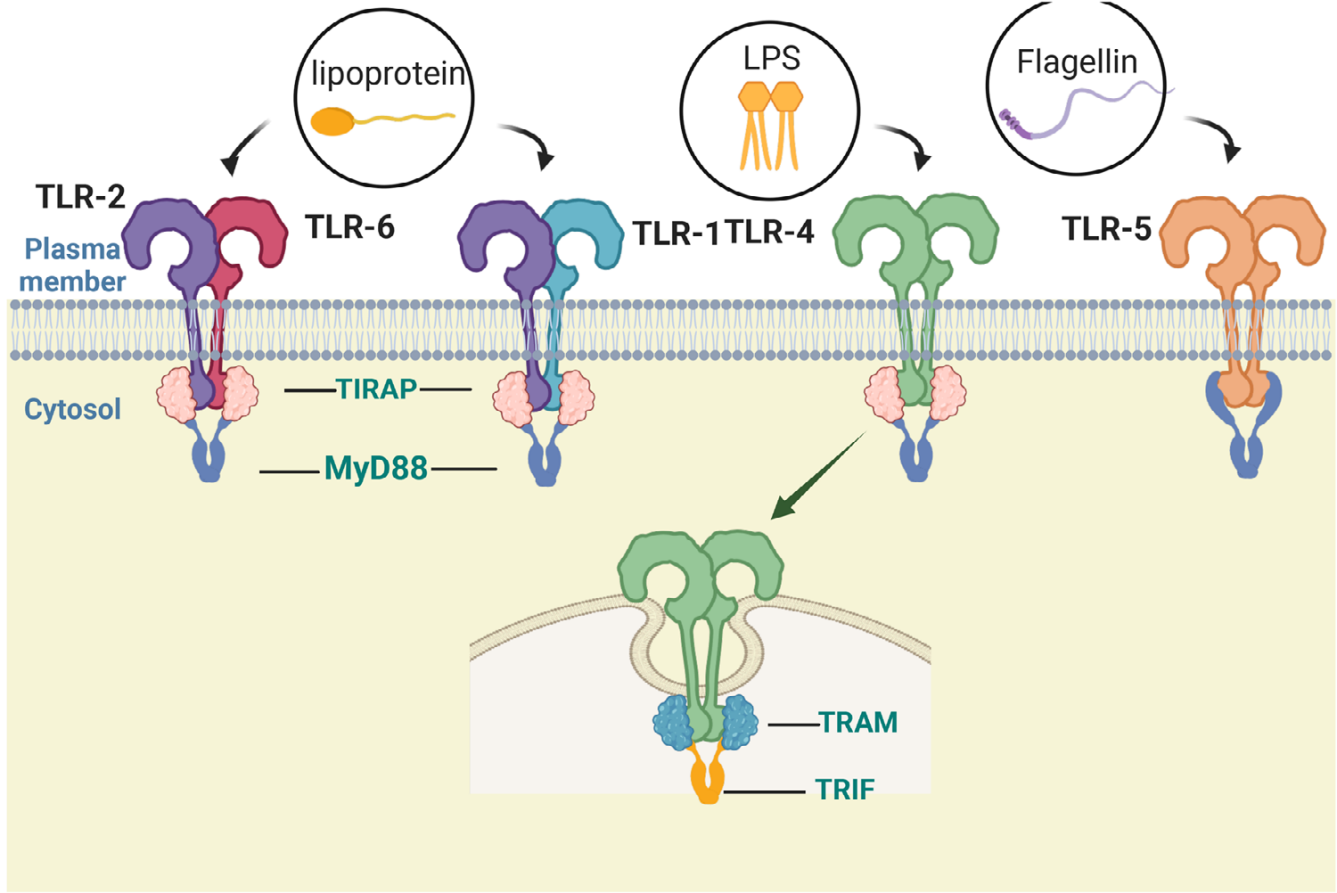
Cell surface TLRs and associated ligands. TLR1,TLR2, TLR4, TLR5, and TLR6 are mainly located on the cell surface. TLR2 usually forms a heterodimer with TLR1 or TLR6 and is involved in the recognition of a variety of PAMPs derived from bacteria, fungi, parasites, and viruses, such as lipopeptides from bacteria. In addition to primarily recognizing LPS on the cell surface, TLR4 can be internalized and retained in the endosome. TLR5 detects flagellin (a component of bacterial flagella).

TLRs on the cell surface, including as TLR1, TLR2, TLR4, TLR5, TLR6, and TLR10, are mainly responsible for identifying lipids, lipoproteins, and proteins found in microbial membranes. Mammals’ TLR2 forms homodimers or heterodimers with TLR1, TLR4, TLR6, and TLR10 to recognize various ligands,^12^, ^13^, ^14^ which together can sense a variety of PAMPs derived from a variety of pathogens, including bacteria, fungi, parasites, and viruses. TLR2 also recognizes a variety of DAMPs generated by necrotic cells, inflammatory processes, and tissue injury.^15^ DCs and macrophages produce a range of proinflammatory cytokines in response to these endogenous ligands of TLRs. Cell surface lipopolysaccharide (LPS) and myeloid differentiation factor 2 (MD2) are recognized by TLR4, which results in the formation of a spatially symmetric M‐type TLR4–MD2–LPS dimer. Gram‐negative bacteria's outer membrane contains a significant amount of LPS, a strong immunostimulatory chemical that can lead to infectious shock.^16^ Apart from LPS, TLR4 is able to identify multiple pathogenic components that activate typical pathways to produce cytokines that promote inflammation and/or IFNs via alternative pathways. For example, the capsid proteins of the virus that causes respiratory infections and the pneumococcal virulent proteins are recognized by TLR4.^17^, ^18^ TLR5 is capable of identifying the continuous structural domain of flagellin.^19^ It is strongly expressed in DCs of the lamina propria of the small intestine, where it detects flagellin from flagellated bacteria, inducing the production of inflammatory factors and thereby modulating innate and adaptive responses to intestinal bacteria (Figure 1).^20^

Inside of cells, regions such as lysosomes, endosomes, and extracellular reticulum (ER) express TLRs, which include TLR3, TLR7, TLR8, and TLR9. They can distinguish between nucleic acids produced by bacteria and viruses as well as self‐nucleic acids found in illnesses like autoimmunity.^21^, ^22^ Additionally, TLRs produce IFNs and inflammation‐related cytokines to trigger antiviral innate immune responses.

**FIGURE 2:**
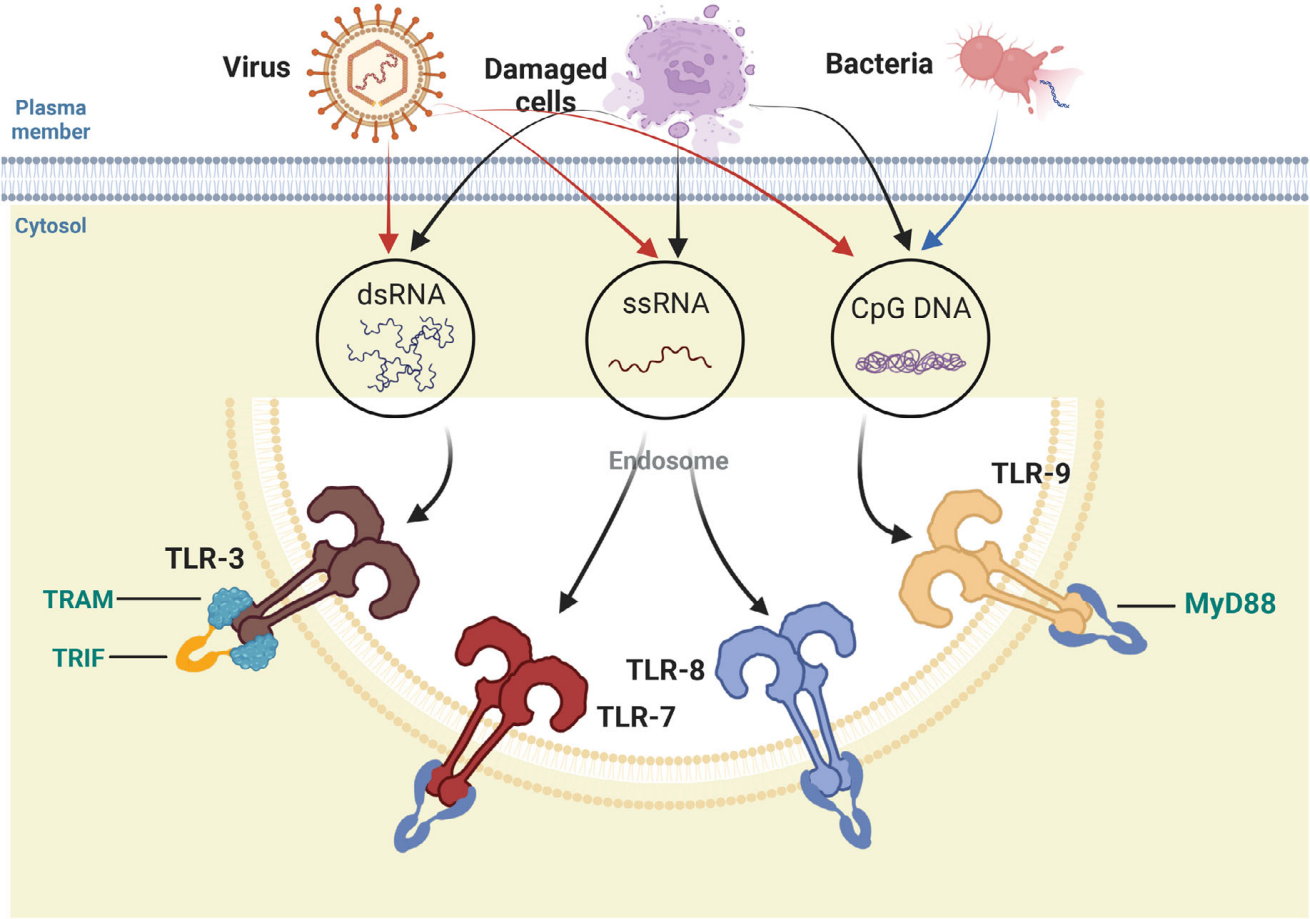
Intracellular TLRs and related ligands. At steady state, TLR3, TLR7, TLR8, and TLR9 are primarily localized to the endoplasmic reticulum and transported to endolysosomes, where they bind their ligands. TLR3 recognizes dsRNA from viruses or damaged cells. TLR7 recognizes ssRNA from ssRNA viruses or damaged cells, and TLR9 recognizes DNA from DNA viruses and bacteria or damaged cells.

TLR3 can identify RNA originating from injured cells, viral double‐stranded RNA (dsRNA), and small interfering RNAs.^23^ When TLR3 is activated, NF‐кB is also activated, which results in the synthesis of IFNs and proinflammatory cytokines.^24^, ^25^ Primarily expressed in plasmacytoid DCs (pDCs), TLR7 identifies single‐stranded (ss) RNA found in viruses and acts as a small purine analog (imidazoquinoline); moreover, it identifies RNA from Streptococcus B bacteria in conventional DCs (cDCs), inducing type I IFN and proinflammatory cytokine production.^26^, ^27^, ^28^ Human TLR8 is responsive to viral and bacterial RNA, and TLR8 is most similar phylogenetically to TLR7. TLR8 is expressed in a variety of tissues, is most highly expressed in monocytes, and is upregulated following bacterial or viral RNA stimulation, as well as inducing IFNs and inflammation‐related cytokine generation.^29^ TLR9 is able to identify DNA from bacteria and viruses that are rich in unmethylated CpG‐DNA motifs. In pDCs, TLR9 plays a major role in the generation of IFN‐α following infection by viruses containing DNA like Herpes simplex virus 1（HSV‐1） and HSV‐2 (Figure 2).^30^, ^31^

The biological roles and ligand recognition of human TLRs 1−9 are well understood. TLR10, on the other hand, is one of the more obscure components within this group, the main limiting factor of which is the lack of suitable mouse models for study, asretroviral insertion into wild‐type (WT) mice does not result in the expression of functional TLR10.^32^ Although TLRs are commonly thought to upregulate proinflammatory cytokine production, recent studies have shown that TLR10 is the only known member of the TLR family that can induce anti‐inflammatory effects.^33^, ^34^ Researchers have suggested that its molecular mechanism of anti‐inflammatory activity may involve competing with other TLRs for ligands or activating the production of the anti‐inflammatory cytokine.^35^

TLR11, 12, and 13 are present in mice but not in humans and are still poorly understood among researchers. TLR11 exists inside the endolysosome and is capable of identifying filamentous protein‐like molecules originating from *Toxoplasma gondii* as well as flagellin or other unknown protein components of UPEC.^36^ Similar to TLR11, TLR12 appears primarily in bone marrow cells and is capable of identifying filamentous proteins from *T. gondii*.^36^ TLR13 is capable of identifying bacterial 23S rRNA and regulates the innate immune response.^37^, ^38^, ^39^

## 3 TLR‐RELATED SIGNALING PATHWAYS

Every TLR that is produced by host cells is made in the ER, moved to the Golgi complex, and then translocated to the intracellular spaces or the cell membrane. UNC93B1 (Unc‐93 homolog B1) is a multichannel transmembrane protein that controls and regulates the translocation of intracellular TLRs (i.e., TLR3, TLR7, TLR8, and TLR9) to endosomes.^40^, ^41^ All NA‐sensing TLRs require the 12‐fold transmembrane protein UNC93B1 to leave the endoplasmic reticulum and travel to endosomes.^42^, ^43^, ^44^ TLR4‐associated protein (PRAT4A) is an additional ER‐resident protein that regulates TLR1, TLR2, TLR4, TLR7, and TLR9 translocation from the ER to the endosomes and plasma membrane.^45^ gp96 (a member of the heat shock protein [Hsp]90 family) in the ER is a universal chaperone for most TLRs.^46^

After recognition of the corresponding ligand through interaction with the LRR, a single TLR recruits members of a group of adapters containing TIR domains in a differential manner and triggers different signaling cascades. The MyD88‐dependent pathway and the TIR domain‐containing adapter‐inducing interferon β (TRIF)‐dependent pathway are the two main categories into which the TLR signaling pathways can be separated.^47^, ^48^, ^49^, ^50^

### 3.1 MyD88‐dependent pathway

With the exception of TLR3, all TLRs influence inflammatory responses by driving NF‐κB and mitogen‐activaaated protein kinases(MAPK) activation via MyD88. In addition to the TIR domain, MyD88 also has a death domain (DD).^48^, ^51^ Moreover, IL‐1R‐associated kinase (IRAK) 4 and MyD88 interact.^52^, ^53^ The defining kinase in the TIR signaling pathway, IRAK4, is a serine/threonine kinase with an N‐terminal DD. It is also the first enzyme to be recruited to the Myddosome complex via TRIF/TRIF‐related adaptor molecule(TRAM) or MyD88/TIR domain‐containing adaptor protein(TIRAP).^53^, ^54^ To cause fast and prolonged activation of NF‐κB, respectively, IRAK4 is first activated, followed by the sequential activation of IRAK1 and IRAK2.^55^, ^56^ Furthermore, TRAF receptor‐associated factor 6 (TRAF6), a protein belonging to the TRAF family that is known to stimulate the NF‐κB pathway, has been demonstrated to interact with IRAK1.^57^, ^58^ TRAF proteins primarily mediate inflammatory responses on the cell surface and through intracellular PRR signaling pathways and specifically drive type I IFN responses.^59^ It has been demonstrated that TRAF6 is downstream of the NF‐κB, p38 MAPK, and JUN N‐terminal kinase (JNK) signaling pathways. The kinase cascades, bridging proteins, and ubiquitination reactions involved in these signaling pathways are now well described. With the exception of TLR3, which only uses the TRIF‐dependent pathway to promote the production of proinflammatory cytokines, all TLRs, including TLR7, TLR8, and TLR9, rely on the MyD88‐dependent pathway.^60^

### 3.2 TRIF‐dependent pathway

**FIGURE 3:**
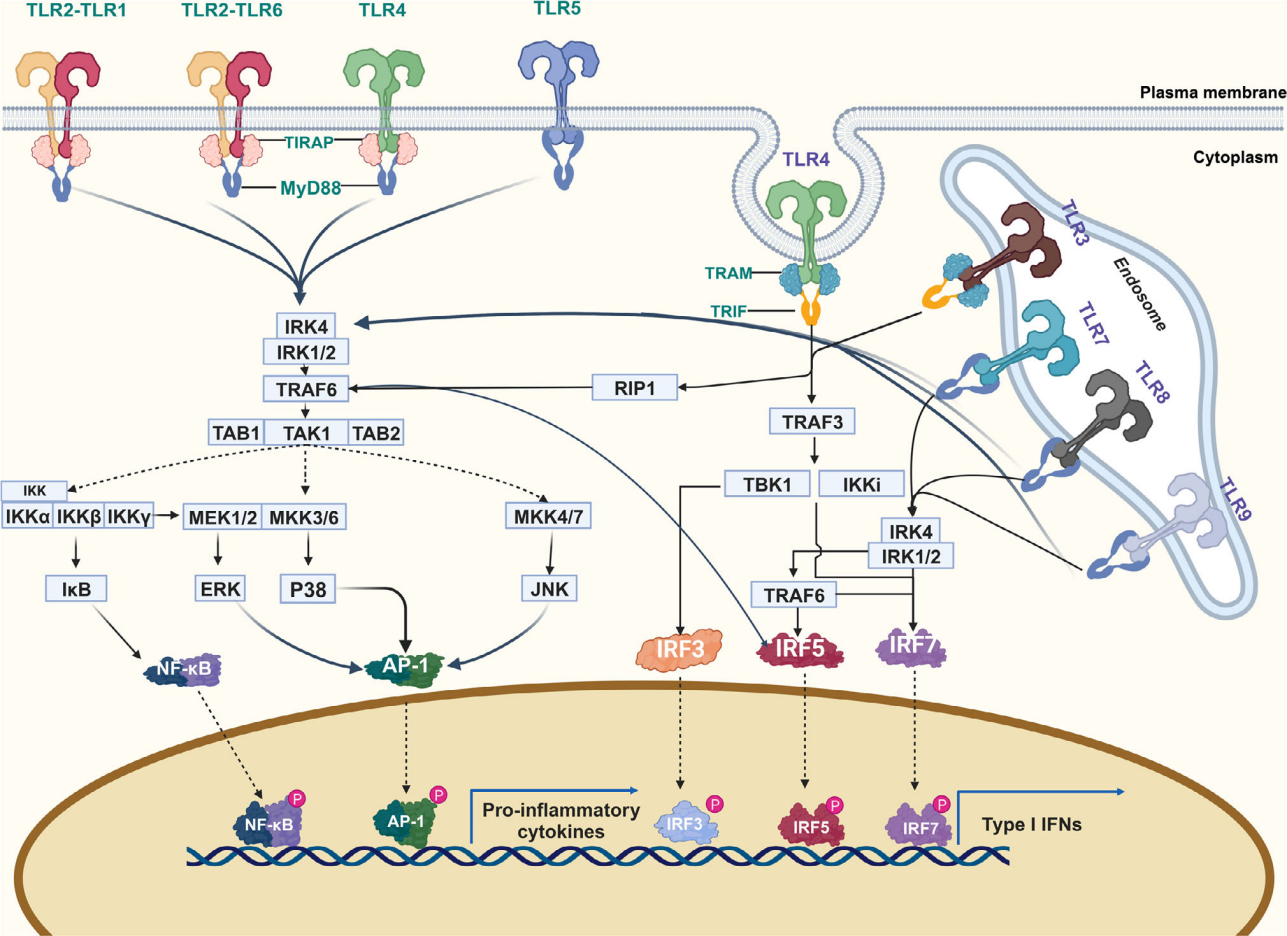
TLR signaling pathways. TLRs signaling can be divided into MyD88‐dependent and MyD88‐independent (TRIF‐dependent) signaling pathways. TLRs are located at the plasma membrane or endosomal membrane and are activated by binding to their ligands, leading to receptor dimerization and recruitment of adaptor proteins such as MyD88, TIRAP, TRIF, and TRAM. IRAK and TRAF6 stimulate TAK1 to activate the IKKγ complex, which further releases NF‐κB into the nucleus. Activated TAK1 also promotes MAPK activation, which in turn stimulates nuclear translocation of AP‐1. Both pathways support proinflammatory cytokine transcription. The MyD88‐independent pathway is activated by TLR3 and TLR4. The TIR domain of TLR recruits TRIF to form complexes containing TRAF3, TBK1, and IKK, which promote nuclear translocation of IRF3 or initiate late NF‐κB through interaction with RIP1 and subsequent TRAF6 activation. Both signal transduction stimulates type I interferon production.

TLR3 recruits TRIF, another adaptor protein, in response to dsRNA stimulation. This activation of interferon regulatory factor 3(IRF3) and NF‐κB transcription factors results in the expression of genes encoding IFNs and cytokines that cause inflammation.^61^ TLR4 and another adapter, TRAM, are required to activate TRIF, and TRAM–TRIF triggers the production of proinflammatory cytokines and IFN.^47^ Through a TRAF‐binding motif located in its N‐terminal region, TRIF binds to TRAF3 and TRAF6, initiating alternate pathways that lead to IRF3, NF‐κB, and MAPK.^62^, ^63^, ^64^, ^65^ In addition, TRIF contains the C‐terminal receptor‐interacting protein (RIP) homotypic interaction motif, and TRIF also interacts with TRAF6 and RIP1, which is responsible for the activation of NF‐κB (Figure 3).^66^, ^67^

## 4 TLR‐MEDIATED ADAPTIVE IMMUNE STIMULATION

TLR activation in innate immune myeloid cells guides lymphocytes to produce the most suitable effector response to eradicate the infection and offers information about the type of invasive pathogen being identified.^68^ The development of DCs is essential for the start of an adaptive immune response.^69^ TLR activation increases the surface expression of costimulatory markers like CD80 and CD86 as well as major histocompatibility complex II(MHCII), which causes DCs to develop into potent antigen‐presenting cells (APCs).^70^, ^71^ For instance, when endosomal TLR3 identifies cells contaminated with viruses that have been swallowed, DC expression responds to the viruses by producing IFNs and interleukin‐12(IL‐12).^72^

TLRs expressed on DCs identify bacteria or virus particles, which are subsequently taken up by the pathogen and phagocytosed or endocytosed. MHC molecules are then used to present the microbial antigen to T cells. This expression takes place in the backdrop of many TLR‐induced signals necessary for the activation of naïve T cells.^73^, ^74^ TLR can also have a direct impact on T and B lymphocyte function. When T lymphocytes are directly stimulated by TLR2 in the absence of APC, regulatory T cells proliferate.^75^, ^76^ In addition, B cell responses and antibody production are also regulated by TLRs.^77^ The production of natural immunoglobulinM(IgM) antibodies and canonical and noncanonical NF‐κB signaling are induced by intrinsic B cell TLR activation, which also promotes B‐cell proliferation. These processes are crucial for defending against bacteria and viruses like influenza.^77^, ^78^ Similarly, KIR3DL2 helps TLR recognize PAMP in NK cells, which in turn triggers a powerful immune response that eliminates the infection. NK cells eliminated infections by overexpressing NKp46, NKp30, and NKG2D on NK cells when TLR2 was activated.^79^, ^80^, ^81^

Not surprisingly, the TLR signaling pathway is tightly controlled, with multiple negative regulators of TLR signal transduction present at different levels to ensure that immune homeostasis is maintained.^82^ IRAK‐M, Toll‐interacting protein, and cytokine signal transduction Inhibitor 1 (SOCS‐1) are examples of interacting TLR signaling pathway inhibitors.^83^, ^84^, ^85^, ^86^

## 5 TLR‐RELATED DISEASES

**FIGURE 4:**
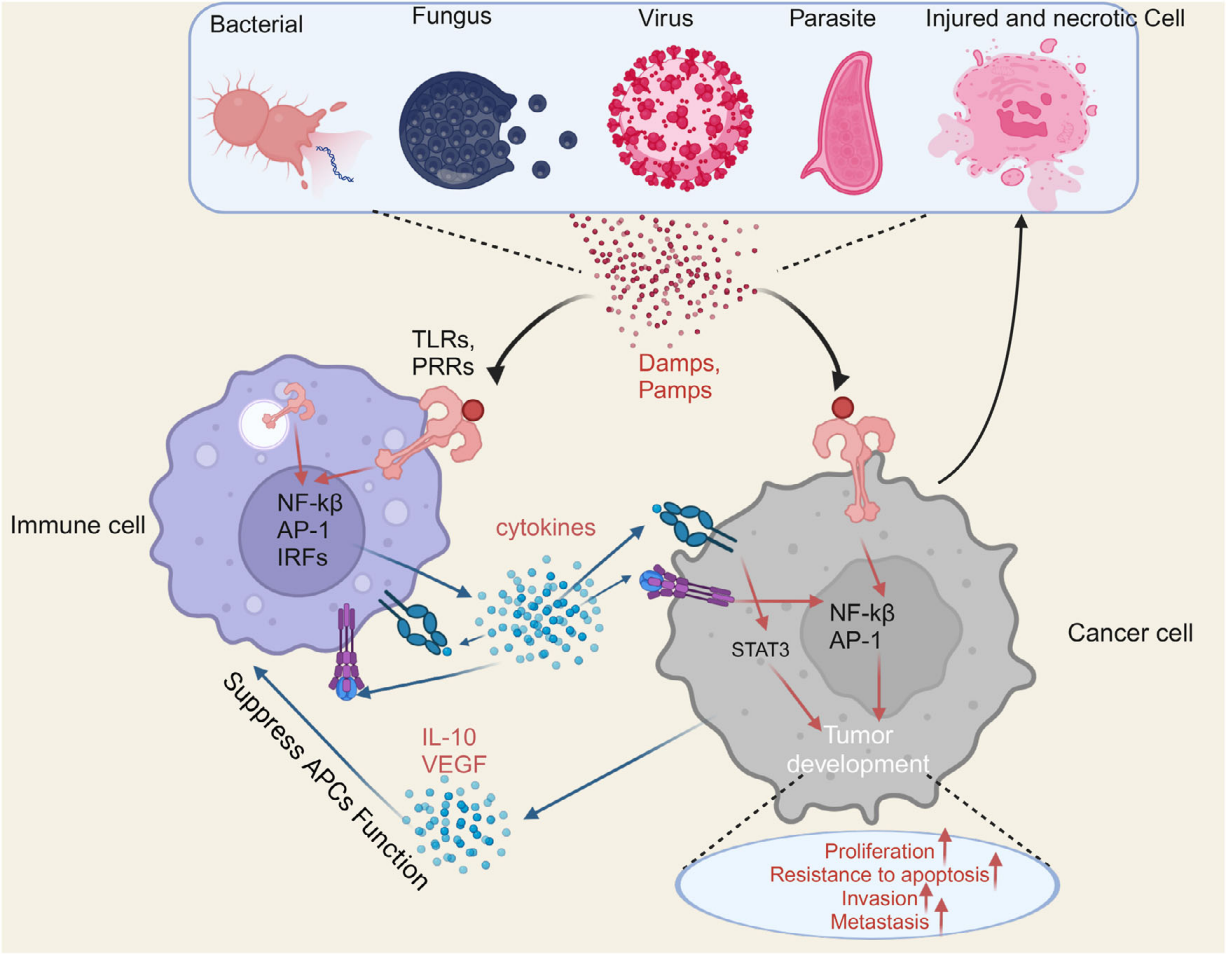
Interactions of TLRs with inflammation and tumor. TLRs are expressed on a variety of cells, including tumor cells and immune cells. TLRs signaling is involved in the carcinogenesis of the tumor microenvironment. TLRs expressed on immune cells and tumor cells are subjected to activation by pamps, damps from microorganisms, viruses, parasites, injured and necrotic cancer cells.The release of cytokines and chemokines by these activated cells is an important part of the tumor microenvironment. Moreover, cytokine‐activated infiltrating immune cells can subsequently induce additional cytokines that impair the function of APCs, leading to tumor immune tolerance.

TLR activation triggers an inflammatory response, which is a defensive response. TLRs play a crucial function in mammalian defense against infections caused by bacteria, they are also engaged in tissue regeneration and repair.^87^, ^88^ However, due to the failure of the regulatory mechanism of TLR signaling, improper activation of TLR signaling may disrupt homeostasis by forming a feedback loop of inflammatory cytokine secretion^,^ inducing the development of inflammation‐associated and autoimmune disorders,^89^ and creating a favorable microenvironment to promote carcinogenesis.^90^ Persistent inflammation creates a favorable microenvironment that contains macrophages, DCs, natural killer cells, T lymphocytes, and B lymphocytes in addition to the surrounding substrate. Such many cells interact with one another directly or through the release of cytokines and chemokines, which affects the development and progression of tumors (Figure 4).^91^, ^92^

### 5.1 Respiratory diseases

Due to its continuous gas exchange function, the lungs are easy target organs for airborne pathogens, allergens, and other toxic substances that cause lung infection or inflammation. The intensity and duration of exposure to harmful substances vary, and lung injury may be acute or chronic.^93^ During the course of the Corona Virus Disease 2019(COVID‐19) study, TLRs were discovered to potentially be important in the illness.^94^, ^95^, ^96^

Zheng et al.^97^ analyzed TLR expression as well as downstream bridging protein expression in COVID‐19 patients with varying stages of the disease. Subsequently, the authors constructed an in vivo infection model and reported that the administration of TLR2 inhibitors after infection modestly improved survival in mice and significantly reduced the release of proinflammatory cytokines such as IL‐6, tumor necrosis factor(TNF), and IFNs, indicating that TLR2‐mediated inflammation is pathogenic in severe acute respiratory syndrome coronavirus 2(SARS‐CoV‐2) infection.^97^, ^98^, ^99^ In a mouse model of coronavirus infection, the TLR3 pathway is induced to stimulate the production of IFN‐β in macrophages and thus impede the development of infection.^100^ In monocyte macrophages and DCs, additional TLR7/8 recognize ssRNA fragments in the SARS‐CoV‐2 genome, inducing a strong proinflammatory response leading to acute lung injury and even death.^101^, ^102^ TLR4 plays a pathogenic role in persistent pulmonary fibrosis under the stimulation of DAMPs. Small‐molecule materials can selectively target MD2/TLR4, destroy the MD4/TLR2 complex, inhibit TLR4 signaling in fibroblasts, and prevent the continuous development of fibrosis. Therefore, appropriate use of TLR inhibitors can effectively prevent and reverse the occurrence and development of lung‐related diseases.^103^, ^104^, ^105^

### 5.2 Cardiovascular diseases

Chronic inflammation of the vascular system is triggered by endothelial dysfunction accompanied by the involvement of multiple risk factors.^106^ TLRs are associated with the atherosclerotic process.^106^, ^107^, ^108^, ^109^, ^110^, ^111^, ^112^ In a vascular injury model, knockout of TLR2 repressed the release of inflammatory cytokines and reactive oxygen species (ROS) in damaged vessels and reduced the formation of neointima, indicating that TLR2 has a crucial role in regulating vascular inflammation and neointima formation following vessel damage. One possible treatment target that shows promise is TLR2 inhibition.^113^, ^114^, ^115^ Poly(I:C) (a TLR3 agonist) activated yes‐associated protein 1(YAP1) through PP1A‐mediated inhibition of MOB1 and large tumor suppressor 1(LATS1) and inactivation of AMPK. Activated YAP1 increased the expression of miR‐152, which inhibited the expression of P27 Kip1 and DNMT1 and caused the growth of neonatal cardiomyocytes. Furthermore,TLR3 activation can also protect the vascular wall.^116^, ^117^ TLR4 is expressed at low levels by endothelial cells in normal vessel walls but is increased in atherosclerotic plaques. The most well‐characterized TLR in the etiology of hypertension is TLR4, which is also important in myocardial inflammation.^118^, ^119^, ^120^, ^121^ To improve myocardial cell survival, TLR4 inhibitors disrupt TLR4‐related TAK‐242/MyD88/NF‐κ B signal transduction, produce proinflammatory cytokines, and decrease inflammatory corpuscle NLRP3 activation.^122^ Activation of TLR4–MyD88 signaling by LPS enhanced mesenchymal stem cell proliferation and prevented cardiomyocyte apoptosis in vitro. LPS pretreatment prior to infusion of mesenchymal stem cells(MSCs) into the heart helps restore in vitro cardiac function.^123^ The flagellin–TLR5–Nox4 axis triggers vascular smooth muscle cell migration and the formation of atherosclerotic plaques.^107^ Activation of TLR9 accelerates the transition of macrophages to foam cells through the NF‐κB and IRF7 pathways.^124^ Researchers have used angiotensin II (Ang II) to increase human plasma cfDNA and found that cell‐free DNA (cfDNA)–TLR9 signaling stimulates macrophage proinflammatory activation and promotes the progression of vascular inflammation as well as atherosclerosis.^106^, ^125^ These findings suggest that TLRs are strong inducers of oxidative stress and endothelial dysfunction, and that TLR‐specific inhibition may have an effect on the management of many illnesses.

### 5.3 Digestive diseases

Hepatic steatosis, which is the initial stage of nonalcoholic fatty liver disease, is followed by inflammatory nonalcoholic steatohepatitis (NASH) and end‐stage liver disease. The disease advances gradually along this spectrum.^126^, ^127^ Compared with those in the control group, patients with simple steatosis exhibited low expression of TLR9 on T cells, which affected intrahepatic CD4+ T cells and peripheral CD4+ and CD8+ T cells and decreased production of proinflammatory factors by T cells. Researchers speculate that there may be adaptive protection against hepatocellular injury, as patients with NASH exhibit similar expression of TLR9 and increased expression of proinflammatory factors (IFN‐γ).^128^

TLRs are widely expressed in intestinal ECs (IECs), DCs, and Møs,^129^ and these cells recognize relevant molecular patterns through TLRs to maintain mucosal immune homeostasis in the intestine.^130^ Ulcerative colitis (UC) is a chronic and recurrent inflammatory disease of the intestine.^131^ According to research by Ruan et al.,^132^ intestinal Roseburia intestinalis flagellin stimulated TLR5‐mediated immune responses and encouraged the production of anti‐inflammatory molecules in IECs, while also lowering TLR5 expression in colitis‐stricken animals. Moreover, R. intestinalis’ butyrate synthesis boosted TLR5 gene expression and reduced colitis. Emodin was employed by other researchers to inhibit the TLR5/NF‐κB signaling pathway, thereby shielding animals against colitis caused by DSS.^132^, ^133^ UC patients severity of inflammation and the correlation between the intestinal TLR9 expression, TLR9 agonists cobitolimod use, reduced the number of Th17 cells, and increased IL10 Tregs, thus correcting the imbalance of Th17/Treg, may induce macrophages into “M2” phenotype, Change the balance of intestinal cytokine on disorders.^134^

Necrotizing small bowel colitis (NEC) results from excessive intestinal epithelial signaling due to TLR4 activation, which is more common in preterm infant bowels than in term infant bowels. Activation of TLR4 by LPS in the lumen leads to intestinal mucosal destruction, and TLR4 activation in the endothelium leads to vasoconstriction and intestinal ischemia, which are characteristic of NEC. Sodhi et al.^135^ used direct inhibition of TLR4 by HMOs (a class of indigestible carbohydrates present in breast milk) to prevent NEC.

Acute pancreatitis (AP) is a pancreatic inflammatory condition marked by asepsis that ultimately results in alveolar cell necrosis. TLR9 is expressed in pancreatic ductal and endothelial cells and resident immune cells (mainly macrophages). Early in the experimental AP process, host genomic DNA is significantly elevated in the blood, and TLR9 protein expression is subsequently upregulated following AP. Moreover, the use of CpG‐ODN1826 (a TLR9 agonist) exacerbates pancreatic injury in rats and increases TNF‐α expression.^136^ Inhibiting TLR9 expression may provide protection against pancreatic injury and hepatocyte injury after AP. However, appropriate use of TLR agonists can alter the imbalance in the intestinal cytokine balance in patients with colitis. TLR inhibition has an important role in the treatment of sepsis and improving the survival rate of septic patients.

### 5.4 Endocrine diseases

Diabetes mellitus (DM) is a widespread metabolic illness syndrome that is becoming more and more common everywhere in the world. The incidence of complications unique to diabetes has significantly increased along with the number of individuals with diabetes and the length of time they have had the disease.^137^ Low‐grade systemic inflammation and immune system disorders are common features of diabetes and related complications.^138^ In patients with diabetes, TLR2, TLR4, and TLR7 expression levels are four to six times higher.^139^, ^140^, ^141^ Guo et al.^139^ showed that TLR2, through activation of NADPH oxidse2(NOX2), can increase endothelial nitric oxide synthases(eNOS) uncoupling and total superoxide production in aortic endothelial cells, leading to impaired NO bioavailability and insufficient endothelium‐dependent vascular relaxation in type 2 diabetes mellitus (T2DM) patients and that knockdown of TLR2 can correct these pathological responses. Targeted TLR2, therefore, may be a new strategy to treat T2DM and cardiovascular complications. In the absence of Tlr7, B cells express PD‐L1 levels and inhibit the CD4 T cells. It prevents diabetes from developing and restricts the growth of CD8 T lymphocytes that are specific to antigens.^140^ Liu and others^142^ have confirmed that Tlr9−/− NOD mice to promote development and beta islet cell differentiation, result in impaired glucose tolerance, insulin sensitivity, prevent type 1 diabetes.

Many TLRs are involved in the complications associated with diabetes. Upregulation of TLR7 is one of the risk factors for the progression of diabetic retinopathy (DR) and consistent with the above findings, TLR7 deletion reduces the release of proinflammatory cytokines. Researchers have used TLR inhibitors to reduce inflammation‐induced retinal damage.^143^, ^144^ Blocking the TLR signaling pathway in advance seems to have excellent therapeutic effects on the occurrence and development of diabetes.

### 5.5 Sepsis

Sepsis, which is characterized by significant systemic inflammation and coagulation activation, is an infection‐induced dysregulation of the host's inflammatory response that results in multiple organ damage and failure, as well as eventual death.^145^, ^146^, ^147^ Because of its high stability, remarkable repeatability, and broad applicability, the cecum ligation and puncture (CLP) sepsis model is widely regarded as the gold standard for research on sepsis.^148^

Adult patients may be at risk for sepsis if they have the TLR2 genotype.^149^, ^150^ Researchers examined the expression of chemokines and inflammatory cytokines in the spleen of WT and TLR2‐deficient mice used in a CLP model. Compared with WT mice, TLR2−/− mice showed lower IL‐10 levels and decreased cysteine asparaginase 3 activation. Additionally, TLR2 had a substantial immunosuppressive effect on the spleen due to sepsis.^151^ Furthermore, in patients with acute myeloid leukemia, mRNA expression of TLR2 and TLR4 was found to be considerably higher in those with sepsis than in those without symptoms of sepsis prior to induction chemotherapy.^152^

The severity of the sepsis disease, inflammatory cytokines, and 28‐day mortality were all positively connected with TLR3 expression.^153^ Anti‐TLR3 antibodies were employed by researchers to reduce the release of inflammatory chemokines. Anti‐TLR3 antibodies considerably decreased sepsis‐induced mortality in animals and mitigated tissue damage brought on by intestinal ischemia.^154^ The results demonstrated that the CLP surgery group had considerably higher levels of TLR2, 4, and 9 mRNA and protein expression than the sham surgery group. Moreover, the activation of these TLRs increased the production of cytokines and the mortality rate of CLP‐induced ALI animals.^155^, ^156^

HDCA (TLR4 antagonist), which controls systemic inflammation, lessens organ damage, and increases survival in septic mice, inhibits the formation of the LPS/TLR4/MD2 ternary complex and prevents the activation of the TLR4 downstream signaling pathway in macrophages.^157^ Chlorthalidomide mitigates sepsis‐induced oxidative stress, mitochondrial structural damage and dysfunction, and cardiomyocyte apoptosis by controlling macrophage polarization via TLR4/NF‐κB/MAPK signaling.^158^ RKH binds to TLR4 directly and inhibits TLR4 activation in immune cells, preventing organ damage and death brought on by sepsis. It considerably lowers sepsis‐induced inflammatory cell activation and overproduction of proinflammatory factors, and it protects against sepsis‐induced death and organ damage.^159^ In LPS‐treated rats, ferrostatin‐1 ameliorates sepsis‐induced cardiac dysfunction and dramatically lowers TLR4, NF‐κB, and IκBα levels.^160^

Through the activation of TLR5, flagellin reduces the production of IL‐1RN, so reducing the severity of sepsis, increasing bacterial clearance, decreasing organ inflammation and injury, and reducing immune cell apoptosis following experimental sepsis.^161^ Sepsis is caused by microbial infections that generate PAMP and/or DAMP. These molecules communicate through platelet‐TLR7, participate in downstream platelet activation, and help form platelet–leukocyte aggregates, which in turn cause thrombocytopenia in sepsis patients.^162^ Mice lacking TLR7 have reduced systemic cytokine production, lower acute kidney injury and bacterial load, and higher survival rates following a range of microbial illnesses when compared with WT mice. Compared with WT mice, mice lacking TLR7 have lower levels of systemic cytokine production, acute renal injury, and bacterial load. They also have higher survival rates following infection with a range of pathogens.^163^ The TLR9 rs187084 and rs352162 polymorphisms can be utilized to assess the risk of sepsis and multiple organ failure in people who have had severe trauma.^164^ When compared with the normal group, the sepsis group's serum TLR9 levels were statistically lower.^165^ Inhibitory CpG sequences that block TLR9 aggression were injected into WT mice to protect them from CLP,^166^ and blocking the TLR9–ER stress signaling pathway lessened the damage that neutrophil extracellular trap (NET)‐induced intestinal EC death caused.^167^

### 5.6 Relationships between TLRs and carcinogenesis

Tumors express a variety of functional TLRs, and the significance that TLR signaling plays in carcinogenesis varies according on the kind of cancer cell. Consequently, the following describes the expression and functions of several TLRs in various malignancies (Table 1).

**TABLE 1:** The role of different TLRs in different diseases.

TLRs	Disease type	Mechanisms
TLR2	SARS‐CoV‐2	↑Proinflammatory cytokines (IL‐6, TNF‐α, and IFN‐γ)^97^, ^98^, ^99^
	Vascular damage	↑Proinflammatory cytokines (TNF‐α, IL‐1β, IL‐6, and MCP‐1) and ROS^113^, ^114^, ^115^
	Oral squamous cell carcinoma	↑Tumor cell growth through extracellular signal‐regulated kinase 1/2^170^, ^171^, ^172^, ^173^
	Breast cancer	↑Neutrophil immunosuppression ↓Tumor cell apoptosis^179^
	Breast cancer	↑Cancer cell migration^180^
	Colorectal cancer	↑Proinflammatory cytokines and ROS^190^
	Colorectal cancer	↑Tumor cell apoptosis^192^
	Gastric cancer	↑Tumor cell migration and invasion^198^
	T2DM	↑Decoupling of eNOS and total superoxide^139^
TLR3	Oral squamous cell carcinoma	↑Tumor cell migration and invasion^202^
	Colorectal cancer	↑Tumor cell death through UNC93B1–IFN‐β^213^
	Colorectal cancer	↑IFN‐γ ↑Cytotoxic immune cells (NK + T cells)^214^
TLR4	Oral squamous cell carcinoma	↑Tumor cell migration through epithelial–mesenchymal transition^220^, ^223^
	Breast cancer	↑Tumor cell adhesion, spread, invasion, and potential growth.^176^, ^224^, ^225^, ^226^, ^227^, ^228^, ^229^
	Breast cancer	↑Tumor cell metastasis through PI3K/Akt/GSK3β /β‐catenin^228^
	Colorectal cancer	↑Tumor cell proliferation through the p‐PAK1/p‐β‐catenin S675 cascade^239^
	Gastric cancer	↑Tumor cell adhesion^245^
		↑Immunosuppressive immune cells (M2)^246^
		↑Tumor cell proliferation and migration^248^
TLR5	Breast cancer	↑Tumor cell invasion through migration (epithelial–mesenchymal transition)^264^, ^265^, ^266^
	Colorectal cancer	↑Proinflammatory cytokines (TNF‐α)^271^
	Gastric cancer	↑Tumor cell malignancy induction^275^
	Colitis	↑Proinflammatory cytokines^132^, ^133^
TLR7	Oral squamous cell carcinoma	↑Tumor cell cisplatin resistance^276^
	Colorectal cancer	↓Tumor cell apoptosis^286^
TLR9	Oral squamous cell carcinoma	↑Tumor cell proliferation^173^, ^299^
	Oral squamous cell carcinoma	↑Tumor cell proliferation and metastasis^300^
	Colorectal cancer	↓Tumor cell apoptosis^308^
	Colorectal cancer	↑Tumor cell migration and invasion^309^, ^310^
	Atherosclerosis	↑Proinflammatory cytokines^106^, ^125^

Abbreviations: eNOS, endothelial nitric oxide synthases; IFN, interferon; MCP, monocyte chemotactic protein‐1; NK, natural killer; ROS, reactive oxygen species; T2DM, type 2 diabetes mellitus; TLRs, IL, interleukin; TNF, tumor necrosis factor.

#### 5.6.1 TLR2

Oral squamous cell carcinoma (OSCC) is an aggressive tumor originating from the oral mucosal epithelium with varying degrees of differentiation. The incidence of OSCC varies by country/region, and most of these cancers are associated with risky lifestyle habits, including smoking, excessive alcohol consumption, and betel nut chewing.^168^, ^169^ TLR2 is expressed on keratin‐forming cells of dysplastic epithelia and squamous carcinoma, whereas TLR2 expression in malignant keratinized cells may be associated with apoptosis resistance. In OSCC cells, TLR2 activation led to the production of miR‐146a‐5p and the subsequent suppression of CARD10, which promoted proliferation and protected the cells from apoptosis and cisplatin‐induced cell death. TLR2 stimulates extracellular signal‐regulated kinases 1/2 to enhance the development of human squamous carcinoma cells.^170^, ^171^, ^172^, ^173^ Additionally, blocking TLR2 inhibited tumor growth.^174^

One of the most common malignant tumors to be discovered, breast cancer (BC) is the primary cause of cancer‐related deaths for women globally.^175^ TLR2 is more expressed in BC tissues than in normal tissues in people with BC.^176^, ^177^, ^178^ High expression of serum amyloid A in BC induces neutrophil immunosuppression by stimulating the TLR2/MyD88‐mediated PI3K/NF‐κB signaling pathway. It also triggers p38 MAPK pathway‐related apoptosis resistance and promotes the development of BC.^179^ In addition, Secli et al.^180^ reported that morgana (a cochaperone of HSP90) released from cancer cells coexisted with and bound to HSP90, inducing cancer cell migration via TLR2. Therefore, some researchers have shown that targeting TLR2 receptors has antitumor effects. Via the TLR2/NF‐κB signaling pathway, UV‐inactivated oncolytic herpes simplex virus type 2 (UV‐oHSV2) activates natural killer cells to release IFNs.^181^ When Cordyceps militaris polysaccharide (CMPB90‐1) binds to TLR2, it changes immunosuppressive TAMs. This results in the activation of ERK as well as the release of Ca2+, p38, Akt, and NF‐κB. TAMs polarize from the M2 to the M1 as a result of this mechanism, which has anticancer effects.^182^

The third most common cancer worldwide, colorectal cancer (CRC) affects almost two million individuals annually and has a poor 5‐year survival rate because of delayed diagnosis and ineffective treatment.^183^ It was discovered that TLR2 expression was much elevated in CRC patients, and this had an impact on patient survival. TLR2 stimulation increased the migration, invasion, and proliferation of CRC cells.^184^, ^185^, ^186^, ^187^ Furthermore, the gut flora contribute significantly to the advancement of CRC, and *Fusobacterium nucleatum* enhances the development of CRC by upregulating the expression of inflammatory mediators via TLR2 signaling.^188^, ^189^ In order to promote CRC, *Pseudomonas* fluorescens interacts with TLR2/4 on host cells to cause the generation of ROS, boosts cholesterol biosynthesis by controlling sterol‐regulatory element binding protein 2 (SREBP2), and activates prooncogenic genes and pathways.^190^ In addition, Meng et al.^191^ shown via cellular and animal studies that TLR2 downregulation impeded the growth of patients with sporadic CRC and colitis‐associated cancer, indicating a potential role for TLR2 in the development of CRC. According to Zhou et al.,^192^ EPS116/TLR2/MyD88 signaling phosphorylated c‐Jun and activated JNK, which in turn increased the overexpression of Fas/Fasl, which in turn induced apoptotic signaling and impeded the growth of CRC.

It was difficult to treat GC patients because they usually did not experience any symptoms in the early stages. Researchers found that TLR2 mRNA and protein expression levels were elevated in mouse gastric cancer models, GC cell lines, and gastric tumors in human GC and that TLR2 overexpression was associated with high histologic grade, microvascular invasion, and cell adhesion and was a poor prognostic factor.^193^, ^194^, ^195^, ^196^, ^197^ 25‐Hydroxycholesterol levels are mediated by increasing TLR2, the NF‐κB gene of MMPS expression, which promotes GC cell migration and invasion.^198^ Researchers have shown that activation of TLR2 induces GC cell proliferation and promotes the generation of ROS. The natural phenolic 18β‐glycyrrhetinic acid regulates TLR2 promoter region methylation and inhibits gastric tumorigenesis, GC cell proliferation, and carcinogenesis induced by TLR2 activation.^195^ However, contrary to the above idea that GC induces the depletion of peripheral and tumor‐infiltrating CD8+ T cells, the downregulation of TLR2 in CD8+ T cells may lead to CD8+ T‐cell immune dysfunction by inhibiting the perforin–granzyme pathway. The use of a TLR2 activator (Pam2Csk8) helps to reestablish CD8+ T‐cell activity for the treatment of viral infections and cancer.^199^ Targeting TLR2 may be one of the treatment targets for cancer treatment. Many studies on the above topic suggest that activating TLR2 deserves careful evaluation of its application in cancer therapy, enhancing the growth of cancer cells and migration and inhibiting TLR2 may constitute combined effective strategies for cancer treatment.^200^, ^201^

#### 5.6.2 TLR3

TLR3 expression in OSCC was twofold. On the one hand, the TLR3 signal sequentially activated IRF3 and NF‐κB after poly I:C (a TLR3 agonist) stimulation, which resulted in the release of IL‐6 and C‐C motif chemokine ligand 5(CCL5), as well as the enhancement of cancer cell migration. This suggests that TLR activation encouraged OSCC aggressiveness and invasion.^202^ In contrast to the above findings, TLR3 activation induced an increase of inflammatory cytokines that suppressed cell proliferation, directly induced cell death, and reduced migration of cells in OSCC.^203^, ^204^, ^205^, ^206^ The different results of TLR3 agonists in OSCC may be related to differences in cell types or in the concentrations of the TLR3 agonists used. Therefore, the use of TLR3 agonists in OSCC remains to be evaluated.

Several investigators have shown that stable expression of TLR3 inhibits cell growth in vitro as well as in vivo and negatively regulates the initiation and progression of human BC.^207^ Furthermore, it was found that reduced TLR3 activation might have the most impact on increasing the risk of BC.^208^ Therefore, several researchers have expected to achieve antitumor therapeutic effects through the use of TLR3 activators. Bernardo et al.,^209^ using an imitation of the poly(I:C) drug targeting TLR3, induced IRF3 phosphorylation and caused increased IFN‐β in BC cells. This was possibly achieved through an autocrine/paracrine positive feedback loop, which would be advantageous for the activation of TNF‐related apoptosis‐inducing ligand(TRAIL)‐related death and the TRAIL death pathways responsible for induced cell death to eliminate BC cells.^209^ Huang et al.^210^ loaded hiltonol (a TLR3 agonist) into BC‐derived exosomes. These exosomes demonstrated strong antitumor activity in a mouse model and human organoids of BC by stimulating the activation of in situ cDC1s and subsequently improving the tumor‐responsive CD8 T‐cell response that followed.^210^ In combination with poly(I:C)‐induced apoptosis, nanomaterials synthesized by Ultimo et al.^211^ significantly inhibited suppressed the development and spread of tumors as well as significantly prolonged the longevity of triple‐negative BC (TNBC) mouse models.^212^

When poly I:C is combined with chemotherapy drugs like paclitaxel (PTX) for chemo‐immunotherapy, Zhao et al.^213^ found that poly I:C preferentially activates the TLR3–UNC93B1–IFN‐β signaling axis, which may lead to colon cancer cell death. Conversely, poly I:C and PTX work together to inhibit colon cancer cells from proliferating.^213^ Reovirus, a noncoated dsRNA virus, promotes NK cell activation and enhances NK cell cytotoxicity against CRC cells by activating the TLR3 signaling pathway and inducing IFN‐γ secretion.^214^ The dual effects of TLR3 should be considered in relevant TLR3 clinical trials. TLR3 activation may increase progression in some tumors or in some individuals. However, more study is required to fully understand TLR3's dual function in tumor biology.

#### 5.6.3 TLR4

In OSCC, in addition to increased TLR4 expression, the distribution of TLR4 likewise increased from the basal hominins to the spiny layer.^215^, ^216^ In addition, several researchers have suggested that TLR4 overexpression promotes OSCC development and proliferation and is closely associated with poor invasion and metastasis in oral cancer patients.^217^, ^218^, ^219^, ^220^, ^221^, ^222^ These findings demonstrated a strong correlation between TLR4 and the onset and progression of OSCC. By inducing the NF‐κB signal transduction pathway, the TLR4 receptor activator (LPS) promotes the epithelial–mesenchymal transition (EMT) and increases the migratory ability of OSCC cells.^220^, ^223^

TLR4 expression levels were shown to be substantially connected with tumor size, cell migration, local lymphatic metastasis, histopathological grade, and tumor stage, and they were shown to be higher in BC tissues than in normal breast tissues.^176^, ^224^, ^225^, ^226^, ^227^, ^228^, ^229^ Moreover, the results of Thomas et al.^230^ showed that TLR4 was involved in the rapid uptake of fetuin‐A by tumor cells, contributing to the rapid adhesion of BC cells, cell spreading, invasion, and underlying growth. In addition, Li et al.^228^ stimulated TLR4 activation in human BC cell lines via LPS, triggering β‐catenin signaling through PI3K/Akt/GSK3β and promoting the transcription of downstream β‐linker protein target genes, ultimately leading to BC metastasis.

The expression of TLR4 gradually increased at different stages of CRC development. Because TLR4 is overexpressed in colitis, it may be crucial in the development of intestinal tumors brought on by inflammation, as well as in promoting cell division, invasion, and metastasis and shielding cancerous cells from dying.^184^, ^231^, ^232^, ^233^, ^234^, ^235^, ^236^ In vitro and in vivo CRC cell metastasis may be induced by activating the TLR4‐dependent NF‐κB signaling pathway and LPS.^237^ Thrombospondin 2 activation of TLR4 enhances HIF‐1α‐mediated glycolysis and promotes tumor growth in CRC.^238^ However, by focusing on TLR4, some researchers hoped to inhibit the growth of CRC. Invasive *F. nucleatum* increases the risk of CRC in vivo through the TLR4/p‐PAK1/p‐β‐catenin S675 cascade. On the other hand, *F. nucleatum*‐induced intestinal carcinogenesis and the expression of β‐catenin and cyclin D1 were considerably decreased upon the injection of a TLR4 inhibitor.^239^ In addition, targeting TLR4 signaling with a TLR4 inhibitor (TAK‐242) reduced the number of infiltrating macrophages and decreased the levels of Inflammatory cytokines in the colon, leading to long‐term effects on tumor growth, which can be beneficial for CRC patients.^240^ MiR‐6869‐5p inhibited CRC cells by directly targeting TLR4‐mediated growth and the generation of proinflammatory cytokines.^241^

TLR4 expression gradually increased in normal gastric cardia tissues, cardiac inflammation, and GC cells; TLR4 expression was found to be more abundant in gastric cancer tissues compared with normal control tissues that were adjacent to the cancerous tissues. Furthermore, there was a strong correlation between the expression level of TLR4 and TNM stage, lymph node metastases, and the growth and spread of tumor cells in GC.^242^, ^243^, ^244^ Sangwan et al.^245^ reported that activation of TLR4 by LPS or Gram‐negative bacteria (*Escherichia coli*) significantly increased the adhesion of GC cells to human peritoneal mesothelial cells, and this increase in adhesion could be abolished by inhibiting the TLR4 signaling cascade and the downstream transforming growth factor β activated kinase 1(TAK1) and mitogen‐activated protein kinase kinase1/2(MEK1/2) pathways. In the same vein, TLR4 deficiency at metastatic sites decreases tumor cell adhesion, thereby linking the TLR4 signaling cascade response to enhanced metastatic adhesion and peritoneal spread.^245^ Similarly, Pseudomonas acnes is abundant in GC tissue and promotes GC by promoting M2 polarization of macrophages via TLR4/PI3K/Akt signaling.^246^ In addition, in GC, *Helicobacter pylori* infection significantly induces miR‐18a‐3p and miR‐4286 expression through TLR4/NF‐κB, which is associated with the progression of gastric cancer.^247^ Gastric cancer cell‐derived exosomes induce autophagy and protumor activation through the HMGB1/TLR4/NF‐кB signaling pathway to enhance the growth and migration of GC cells.^248^

Similar to those studies mentioned above, TLR4 has been proven by researchers to be a crucial target for the treatment of GC.^249^, ^250^, ^251^ Yamaguchi et al.^252^ promoted M1 polarization through the TLR4/NF‐кB p65 signaling pathway using PTX. In addition, LPS‐mediated activation of TLR4, the NF‐κB common mediates miR‐18a‐3p and the expression of miR‐4286, increases cancer cell proliferation and motility, and inhibits the expression of BZRAP1, all of which lead to in vitro tumor progression. Researchers have shown that TAK‐242 selectively binds to TLR4, disrupts the interaction between LPS and TLR4, and inhibits downstream signaling pathways, which is effective in treating *H. pylori*‐associated gastric cancer.^247^ Zhuang et al.^253^ expected that targeting TLR4 would convert M2 macrophages to M1 macrophages and alter the tumor microenvironment (TME) to achieve therapeutic effects on tumors. Sophoridine inhibited M2‐TAM polarization, increased M1‐TAM polarization via the TLR4/IRF3 pathway, inhibited infiltration of TAMs by downregulating chemokine C‐Motif receptor 2 expression in the GC microenvironment, and ultimately improved the cytotoxic capabilities of CD8+ T cells while reducing CD8+ T‐cell failure. Sophoridine reshaped the immunological milieu of GC and had therapeutic effects on tumors by acting on macrophages and CD8+ T cells.^253^ Researchers, through exciting roles and antagonistic effects, can manipulate TLR receptors related to tumor treatment and intervention. Many researchers have chosen to use TLR4 antagonists and found that they significantly inhibit the growth and emergence of tumors.^240^, ^247^ Thus, the conversion of TLR4 activation from antitumor to protumor activity by agonists—which depends on a variety of criteria, including timing, duration, and strength—remains a challenging issue for scientists studying tumor immunotherapy.^220^, ^223^, ^228^, ^245^, ^247^

#### 5.6.4 TLR5

In addition to stimulating reactions of inflammation, TLR5 also activates invasion, migration, and cytokine release in cancerous cells.^254^, ^255^, ^256^ Omar et al.^257^ collected OSCC and squamous cell carcinoma of the cervix (CSCC) samples for comparison and found that The OSCC samples have higher levels of TLR5 than the CSCC samples. The clinical outcome of OSCC was more aggressive than that of CSCC, and this difference was speculated to be related to the differential expression of TLR5 in malignant tumors.^257^ They further found that TLR5 expression levels were also greater in oral cancers than in skin cancers and concluded that TLR5 is usually activated more endogenously in oral cancers.^258^ This difference might be related to high levels of bacterial attachment in the oral cavity,^259^, ^260^, ^261^ where flagellin is a component of the bacterial flagellum anchored at one end of the cell membrane.^262^

According to a study by Chen et al.,^263^ more than 60% of BCs express the TL5 protein. TLR5 overexpression had a positive correlation with lymph node metastasis and a negative correlation with histological grade.^263^ Downregulation of TLR5 in TNBC promoted vascular endothelial growth factor receptor expression and angiogenesis, leading to the proliferation of TNBC cells through the TRAF6 and SOX2 pathways to increase tumor aggressiveness and EMT expression.^264^, ^265^, ^266^

CRC patients exhibit increased TLR5 expression from normal mucosa to adenoma or adenocarcinoma.^267^ Higher TLR5 expression in tumor tissue was linked to a better prognosis for patients with CRC.^268^, ^269^ Additionally, several CRC patients had different survival rates when single nucleotide polymorphisms in the flagellin receptor TLR5 were discovered by researchers. By lowering the IL‐6 levels, rs5744174/F616L may directly promote enhanced CRC survival (Table 1), whereas rs2072493/N592S displayed the reverse pattern.^270^ Thagia et al.^271^ reported that suppressor of cytokine signaling‐3 (SOCS3) promoted an increase in TLR5‐induced TNF‐α, disrupted intestinal epithelial barrier function, exacerbated the inflammatory process, and promoted CRC development.

Kasurinen et al.^272^ showed that high tissue expression of TLR5 could indicate that gastric cancer patients have a better prognosis. Polymorphisms in TLR5 might favor the development of autoimmune atrophic gastritis and GC and are significantly linked to an elevated risk of GC.^273^, ^274^ Terawaki et al.^275^ reported that TLR5 signaling pathway activation induces an increase in IRAK‐1/4 expression and promotes an increase in leukemia inhibitory factor concentration in plasma, which contributes to the induction of cachexia in gastric cancer cells. In addition, activation of living signaling pathways may also participate in changes in cellular functions, like movement, development.^275^ The expression and prognosis of TLR5 in different tumor diseases are different, and the use of agonists or antagonists remains to be further explored.

#### 5.6.5 TLR7

According to research by Mahmoud et al.,^276^ human OSCC cells have functionally overexpressed TLR7, and TLR7 activation may contribute to the development of cisplatin resistance in these cells. According to studies by Ni et al.,^277^ patients with high TLR7 expression in OSCC had poor prognosis and poor differentiation. They also discovered that high TLR7 expression in OSCC has a protumorigenic effect.^277^

Several researchers have hoped to achieve antitumor therapeutic effects in BC patients through the use of TLR7 agonists. On the one hand, researchers have coincidentally chosen to target tumor macrophages. A small‐molecule TLR7 agonist (1V209‐Cho‐Lip) was designed by Wan et al.^278^ that stimulated TAMs to transform into M1‐like macrophages and to produce memory CD8+ T cells, which in turn produced protective immunological memory and shown enhanced anticancer effects. R848 (a TLR7/8 agonist) was loaded into dendrimers to remodel TME for effective cancer immunotherapy, effectively polarizing M2 macrophages to the M1 phenotype, increasing the maturity and activity of APCs, decreasing the amount of immunosuppressive myeloid cells, and enhancing the infiltration of tumor cytotoxic T cells to significantly stimulate the TME.^279^ Moreover, Francian et al.^280^ encapsulated TLR agonists (including R848 and CpG 1826) in C3 liposomes for specific delivery, activated APCs, and induced tumor‐specific adaptive immune responses, resulting in reduced tumor growth in BC models. In addition, treating invasive BC models with an intratumoral TLR agonist (PNP‐R848) retarded tumor growth and inhibited lung metastasis, and other investigators have further enhanced the antitumor effect by loading imidazoquinolines such as R848 as cyclic dinucleotides in biodegradable hydrogels or by tethering TLR7 agonists to oxaliplatin‐based platinum (IV) precursor drugs.^281^, ^282^, ^283^, ^284^, ^285^

Researchers have recently found that the TLR7 and TLR8 genes and proteins are highly upregulated in CRC and are closely associated with cancer cells, but are rarely identified in leukocytes that have infiltrated stromal tumors. Furthermore, it has been demonstrated by other researchers that the persistent activation of TLR7, which is expressed by multipotent CD133+ colon cancer‐initiating cells and tumor cells from CRC, sustains the inflammatory response, facilitates resistance to apoptosis, and encourages the growth of new tumors.^286^ Thus, several investigators have hoped to target TLR7 for antitumor effects and found that TLR7 ligands attenuate colitis‐associated colon cancer.^287^, ^288^ Combining R848 with oxaliplatin, Liu et al.^289^ observed a notable rise in M1‐like macrophages and more efficient tumor development suppression, indicating that R848 remodels myeloid‐derived suppressor cells (MDSCs) and their differentiated phenotype at the tumor site, reversing the immunosuppressive effects of oxaliplatin. The TLR7/8 agonist 3M‐011 is a potent adjuvant for CRC treatment and has significant local and systemic antitumor effects. OMC in combination with ionizing radiation had significant antitumor activity. These effects were mediated by NK cells, which are primarily cytotoxic T cells that require DC activation and are the primary target of TLR7/8 agonists.^290^ In pancreatic ductal adenocarcinoma (PDAC), the use of the R848 amplified the antitumor effect of vaccination by modulating the immunosuppressive TME in PDAC, as seen by an increase in APC maturation, a decrease in regulatory T cells, and an increase in tumor antigen‐specific CD8+ T cells.^291^

Patients with GC who had high TLR7 expression had a better prognosis. The mRNA and protein levels of TLR7 in GC tissues were significantly lower than those in neighboring tissues or normal gastric epithelial tissues.^292^ TLR7 is essential to the immunological milieu of GC and is implicated in the course and prognosis of GC. TLR7 expression was favorably linked with the infiltration of DCs, macrophages, neutrophils, and T lymphocytes.^293^ Furthermore,TLR7 expression was shown to be significantly higher in erosive gastric tissue specimens as compared with controls and to be significantly lower as the disease advanced to gastric cancer, according to Shirafkan et al.^294^ Acute inflammation was significantly impacted by the early disease phase elevation in TLR7 expression, but not chronic inflammation. TLR7 downregulation may, via many pathways, contribute to the development of GC.^294^

Wang et al.^295^ synthesized a GC vaccine by covalently linking a TLR7 agonist to the GC antigen MG7‐Ag quadruple epitope, which inhibited gastric tumor growth and immune tolerance. Ma et al.^296^ constructed a bifunctional small hairpin RNA (shRNA) vector containing a Bcl‐2 silencing shRNA and TLR7‐stimulated ssRNA, and stimulation with this bifunctional vector in vitro promoted significant apoptosis in mouse gastric cancer cells and inhibited subcutaneous gastric cancer cell growth in vivo by regulating the expression of apoptosis‐related proteins and inducing the release of IFNs. The use of TLR agonists to target APCs and activate the induction of adaptive immunity against poorly immunogenic autoantigens is important for improving the efficacy of cancer immunotherapy.^278^, ^279^, ^290^

#### 5.6.6 TLR9

TLR9 is linked to OSCC invasion and may be a major factor in the malignant transformation of the oral mucosa.^173^, ^216^, ^297^, ^298^ Activation of TLR9 in OSCC using CpG‐ODNs stimulated tumor cell proliferation.^173^, ^299^ For the first time, Tuomela et al.^300^ demonstrated that host DNA in chemotherapy‐killed cancer cells was quickly incorporated into cancer cells that survived, which subsequently continued to induce carcinogenesis or metastasis as invasion‐inducing TLR9 ligands.

Compared with those in normal cells, TLR9 in human BC cell lines had the highest intracellular expression, and its aberrant expression in tumor cells might promote tumor growth and invasion.^301^, ^302^ While Singh et al.^303^ reported increased TLR9 expression in patients treated with neoadjuvant chemotherapy (NACT) according to immunohistochemistry results, an analysis of publicly available datasets revealed that elevated TLR9 expression was associated with increased overall survival in NACT‐treated patients. In vitro, triggering TLR9 with a TLR9 agonist (CpG ODN) was found to reduce cell proliferation and alter proinflammatory cytokines, thereby facilitating the inhibition of hormone receptor‐positive BC cells (T47D) and triple‐negative BC cells (MDA‐MB‐468).^304^ Combining a TLR9 agonist (CpG) with a polyspecific integrin‐binding peptide (PIP) to generate a tumor‐targeting immunomodulator, referred to as PIP‐CpG, triggered tumor regression and prolonged the survival of mice with BC tumors. PIP‐CpG converts an immunosuppressive TME dominated by MDSCs into a lymphocyte‐rich TME that infiltrates activated CD8+ T cells, CD4+ T cells, and B cells and leads to a T‐cell‐mediated tumor‐specific immune response.^305^

Gao et al.^306^ successfully constructed an acute colitis–chronic colitis–adenoma–adenocarcinoma model by the AOM/DSS induction method and shown that as colorectal lesions were more severe, TLR9 expression levels rose.^307^ Furthermore, significant correlations were found between high TLR9 expression and poor prognosis, invasion, metastasis, and TNM staging of cancers. Necrotic cancer cells release cfDNA, which increases CRC cell survival by stimulating TLR9 signaling. This results in a decrease in apoptosis and an increase in programmed cell survival.^308^ Additionally, cfDNA could activate TLR9 to initiate downstream MyD88 signaling to promote CRC cell growth and facilitate cell movement and invasion.^309^, ^310^

Wang et al.^311^ reported that TLR9 plays a key role in LPS‐induced NET formation, and a TLR9‐deficient human colorectal cell line (HCT116) cultured in LPS‐induced neutrophil medium exhibited significantly reduced tumor cell proliferation, migration, and invasion. Lindsay et al. coloaded the hydrophobic chemotherapeutic drug doxorubicin (DTX) and Incorporation of cholesterol‐modified TLR9 agonist in synthetic high‐density lipoprotein cholesterol (HDL) nanodiscs, and found that the use of DTX‐sHDL/CpG inhibited tumor growth and prolonged animal life.^312^, ^313^

These findings showed that TLR9 mediates gastric cancer inflammation, is abundantly expressed in GC samples, and facilitates the migration of cancer cells.^314^, ^315^ In addition, the TLR9 rs5743836 and rs187084 polymorphisms were linked to a significant oncogenic risk of gastric cancer.^316^ In GC, *H. pylori* DNA could enhance the growth, migration, and invasion of GC through activation of TLR9.^317^ Varga et al.^318^ reported that patients living in areas at high risk of gastric cancer expressed significantly greater levels of TLR9 in gastric ECs than patients living in low‐risk areas did, and *H. pylori* strains isolated from these patients simultaneously induced greater TLR9 activation.

High TLR9 expression in most human tumors, cancer cell growth, invasion, survival, and metastasis are important factors.^173^, ^297^, ^300^, ^301^, ^302^, ^308^, ^314^, ^315^ However, most studies have shown that TLR9 agonists are promising therapeutic agents for cancer. Even so, their mechanisms of action must be elucidated for maximum therapeutic benefit.^304^, ^305^, ^308^ Moreover, researchers have shown that TLR9 agonists are also highly effective and beneficial when combined with traditional cancer treatment (i.e., radiotherapy or chemotherapy).^312^, ^313^

## 6 CLINICAL THERAPY INVOLVING TLRS

The expression of TLRs is elevated in multiple cancer types, such as those affecting the liver, intestinal tract, and oral cavity. These receptors are essential for the development and spread of malignant tumors as well as the prognosis of cancer.^170^, ^171^, ^172^, ^173^, ^184^, ^185^, ^186^, ^187^, ^202^, ^231^, ^232^, ^233^, ^234^, ^235^, ^236^, ^319^, ^320^ TLRs promote carcinogenesis by inducing different cells to release proinflammatory cytokines and antiapoptotic factors, recruiting immune cells, and promoting cell proliferation in TME, thereby creating a tumor‐friendly environment. Their therapeutic use is expected as well, considering the important function played by the molecules in the TLR pathway in the innate immune system; however, this is probably because immune cells and/or cancer cells activate distinct TLRs and downstream signaling cascades; alternatively, because of the temporal sequence of TLR activation in cancer cells or immune cells, studies on the role of TLR antagonists and agonists in inflammation and even cancer have been controversial (Table 2).^41^, ^321^, ^322^, ^323^

**TABLE 2:** Clinical trials of TLR agonists and antagonists in disease.

TLR agonists	Molecule	Treatment	Application	Phase	Status	NCT Number
TLR1/2	XS15	Combination with vaccine	Advanced solid and hematological malignancies		AVAILABLE	NCT05014607
		Combination with atezolizumab	Fibrolamellar hepatocellular carcinoma	I	RECRUITING	NCT05937295
		Combination with vaccine	Sarcoma	I	RECRUITING	NCT06094101
TLR3	Rintatolimod	Combination with durvalumab	Metastatic pancreatic cancer	I	NOT_YET_RECRUITING	NCT05927142
	BO‐11II	Combination with pembrolizumab	Melanoma	II	ACTIVE_NOT_RECRUITING	NCT04570332
	Poly ICLC	Combination with echopulse standard of care PD‐1 therapy	Melanoma	I/II	ACTIVE_NOT_RECRUITING	NCT04116320
		Combination with drug	Low‐grade B‐cell lymphoma	II	COMPLETED	NCT01976585
		Combination with pembrolizumab	Metastatic colon cancer	I/II	COMPLETED	NCT02834052
		Combination with adjuvant vaccine and surgical resection	Melanoma	I|II	COMPLETED	NCT01079741
		Combination with tremelimumab and durvalumab	Head and neck squamous cell carcinoma	I|II	COMPLETED	NCT02643303
TLR2/4	BCG	Combination with chemotherapy and RFA	Unresectable colorectal liver metastases	I	NOT_YET_RECRUITING	NCT04062721
TLR4	GLA‐SE	Combination with radiation therapy	Soft tissue sarcoma	I	COMPLETED	NCT02180698
		Combination with vaccine	Skin melanoma	I	COMPLETED	NCT02320305
	GSK1795091	Combination with pembrolizumab	Neoplasms	I	COMPLETED	NCT03447314
TLR5	MobilanM‐VM3	Monotherapy	Prostate cancer	I|II	UNKNOWN	NCT02844699
	Entolimod	Monotherapy	Unspecified adult solid tumor, protocol specific	I	COMPLETED	NCT01527136
		Combination with intensity‐modulated radiation therapy Chemotherapy (cisplatin)	Head and neck squamous cell carcinoma	I	WITHDRAWN	NCT01728480
	CBLB50II	Monotherapy	Colorectal cancer	II	UNKNOWN	NCT02715882
TLR7	Imiquimod	Combination with radiation and cyclophosphamide	Breast cancer|metastatic breast cancer|recurrent breast cancer	II	COMPLETED	NCT01421017
		Monotherapy	Breast cancer|breast neoplasms	II	COMPLETED	NCT00899574
TLR7/8	BDB018	Combination with pembrolizumab	Tumor, solid	I	ACTIVE_NOT_RECRUITING	NCT04840394
	BDB001	Combination with pembrolizumab	Tumor, solid	I	ACTIVE_NOT_RECRUITING	NCT03486301
		Combination with atezolizumab	Tumor, solid	I	ACTIVE_NOT_RECRUITING	NCT04196530
		Combination with pertuzumab	Metastatic breast cancer	II	RECRUITING	NCT05954143
	BDC‐1001	Combination with nivolumab	HERII‐positive solid tumors	I|II	RECRUITING	NCT04278144
	R848	Combination with drug	Melanoma	II	COMPLETED	NCT00960752
	Resiquimod	Combination with pembrolizumab	Advanced solid tumor	I|II	RECRUITING	NCT04799054
		Combination with pembrolizumab and drug	Head and neck neoplasms	II	RECRUITING	NCT05980598
	RO71199II9	Combination with tocilizumab	Carcinoma, hepatocellular	I	COMPLETED	NCT04338685
	SHRII150	Combination with chemotherapy PD‐1 or CD47 antibody	Solid tumor	I|II	UNKNOWN	NCT04588324
	STM‐416	Monotherapy	Nonmuscle‐invasive bladder cancer	I|II	RECRUITING	NCT05710848
TLR8	VTX‐II337	Combination with pegylated liposomal doxorubicin hydrochloride or paclitaxel	Malignant ovarian mixed epithelial tumor	I	COMPLETED	NCT01294293
		Combination with cetuximab	Metastatic squamous neck cancer	I	COMPLETED	NCT01334177
		Combination with cisplatin or carboplatin, 5‐FU and cetuximab	Head and neck squamous cell carcinoma	II	COMPLETED	NCT01836029
		Combination with durvalumab and drug	Ovarian cancer	I|II	COMPLETED	NCT02431559
TLR9	MGN1703	Combination with ipilimumab	Advanced cancers	I	ACTIVE_NOT_RECRUITING	NCT02668770
	DVII81	Combination with approved anti‐PD‐1 inhibitor	Advanced non‐small cell lung cancer	I	COMPLETED	NCT03326752
	CPG 7909	Monotherapy	Non‐Hodgkin lymphoma	I|II	COMPLETED	NCT00185965
		Combination with drug	Esophageal cancer	I|II	UNKNOWN	NCT00669292
	SD‐101	Combination with drug and radiation therapy	Follicular lymphoma	I|II	COMPLETED	NCT02927964
		Combination with drug	Advanced malignant solid neoplasm	I	COMPLETED	NCT03831295
		Combination with drug and radiation therapy	B‐cell Non‐Hodgkin lymphoma	I	ACTIVE_NOT_RECRUITING	NCT03410901
		Combination with nivolumab and radiation therapy	Metastatic pancreatic adenocarcinoma	I	COMPLETED	NCT04050085
		Combination with ipilimumab and radiation therapy	B‐cell lymphoma of mucosa‐associated lymphoid tissue	I|II	COMPLETED	NCT02254772
		Combination with nivolumab and ipilimumab	Metastatic uveal melanoma in the liver	I	RECRUITING	NCT04935229
		Combination with pembrolizumab and nivolumab and ipilimumab	Hepatocellular carcinoma	I|II	RECRUITING	NCT05220722
		Combination with pembrolizumab and drugs and stereotactic body radiation therapy	Prostatic neoplasms	II	ACTIVE_NOT_RECRUITING	NCT03007732
	CMP‐001	Combination with nivolumab	Melanoma	II	RECRUITING	NCT04401995
		Combination with radiation therapy and nivolumab and ipilimumab	Colorectal neoplasms malignant|liver metastases	I	COMPLETED	NCT03507699
		Combination with nivolumab	Melanoma	II	ACTIVE_NOT_RECRUITING	NCT03618641
		Combination with nivolumab	Metastatic prostate adenocarcinoma	II	RECRUITING	NCT05445609
		Combination with pembrolizumab surgical procedure	Cutaneous melanoma	II	RECRUITING	NCT04708418
	Tilsotolimod	Combination with ipilimumab and nivolumab	Advanced cancer	I	ACTIVE_NOT_RECRUITING	NCT04270864
Antagonist						
TLR4	TAK‐II4II	Monotherapy	Acute‐on‐chronic liver failure	II	UNKNOWN	NCT04620148
		Monotherapy	Sepsis	III	COMPLETED	NCT00143611
TLR7, 8	M5049	Monotherapy	Dermatomyositis|polymyositis	II	RECRUITING	NCT05650567
		Monotherapy	Systemic lupus erythematosus	II	RECRUITING	NCT05162586
TLR7, 8, 9	IMO‐8400	Monotherapy	Diffuse large B cell lymphoma	I|II	COMPLETED	NCT02252146

Abbreviations: 5‐FU, 5‐Fluorouracil; CD47, cluster of differentiation 47; NCT, nationl clinical trial; PD‐1, programmed death 1; RFA, radiofrequency ablation; TLR, Toll‐like receptor.

Clinical trial data sources: clinicaltrials.gov.

### 6.1 TLR agonists for therapy

The first United States Food and Drug Administration‐approved TLR7 agonist, imiquimod, was used to treat superficial basal cell carcinoma.^323^ During the epidemic, imiquimod was found to provide satisfactory innate and acquired immune stimulation and to help eliminate SARS‐CoV‐2 in the early stages of infection but may cause cytokine storms and persistent inflammation as side effects in the later stages of infection.^324^, ^325^ Researchers used imiquimod to reduce angiotensin‐converting enzyme 2(ACE2) and increase IFN‐β expression to trigger viral resistance mechanisms in human bronchial epithelial cells and subsequently improve viral infection tolerance by reducing the levels of epithelial cytokines induced by viral stimulation involved in severe COVID‐19 infection.^326^

The analysis revealed a consistent decline in the quantity of TLR ligand‐related clinical trials that were started between May 2012 and May 2014. Several researchers have shown that TLR agonists are cancer treatment drugs that can induce immune suppression, disrupting TLR agonist‐induced immune stimulation and thus inhibiting antitumor immune effects. One of the main challenges facing TLR agonists in tumor immunotherapy is the production of immunosuppressive substances. TLR agonists have the ability to destroy cancer cells, but this may not happen unless we suppress negative regulators (including Tregs) or tip the scales in favor of an overpowering proinflammatory response.^327^ After many checkpoint inhibitors (CPIs) were approved for the treatment of melanoma patients,^328^, ^329^, ^330^, ^331^ tumor immunotherapy has sparked a renewed interest among physicians and clinical oncology worldwide.^332^ In previously incurable metastatic patients, checkpoint blockers now allow for long‐lasting clinical responses, revolutionizing the treatment of oncology.^333^ Notwithstanding the advancements, a sizable portion of patients, regrettably, do not respond to CPI therapy or initially respond to immunotherapy before relapsing and progressing, which is when alternative therapeutic approaches started to emerge.^334^, ^335^ Therefore, researchers expect to achieve results in cancer treatment by combining TLR agonists and immune checkpoint inhibitors.^336^, ^337^, ^338^, ^339^

Rintatolimod, a poly I:C‐derived dsRNA molecule, is used in combination with INFα and celecoxib to modulate the serum levels of inflammatory cytokines and chemokines in patients receiving systemic chemokine modulation (CKM).^340^ A phase I clinical study demonstrated that systemic CKM administration reprogrammed the local TME in patients with advanced TNBC to selectively enhance cytotoxic Tlymphocyte(CTL) inward flow, and a phase 2 trial was intended to assess CKM's potential in conjunction with programmed death 1(PD‐1) inhibitors.^341^ Motolimod, a TLR8 agonist, enhances NK cell function and potentiates cetuximab‐mediated ADCC, displaying characteristic adverse event (AE) profiles, such as injection site responses, fever, and chills. A phase Ib clinical trial evaluating the safety and antitumor activity of motomod in combination with cetuximab in the treatment of patients with HNSCC revealed that motomod could be safely used in combination with cetuximab and exhibited encouraging antitumor activity.^342^ Another phase II clinical trial revealed that a significant benefit was observed in patients with HPV‐positive oropharyngeal cancer treated with motolimod and the EXTREME regimen (a combination of standard chemotherapy/cetuximab) in patients.^343^ Synthetic oligonucleotide SD‐101 has a CpG motif. Its AEs include injection site reaction that is responsive to over‐the‐counter agents and mild to moderate influenza‐like symptoms. SD‐101 injected intratumoriously alters the TME in a way that promotes IFNs and CD8+ T‐cell infiltration. When combined with pembrolizumab, these modifications may lead to high response rates, especially in individuals who have not had anti‐PD‐1 therapy before.^344^

### 6.2 TLR antagonists for therapy

TLR activation in inflammatory diseases supports its pathophysiology through abnormal secretion of proinflammatory cytokines and chemokines, which in turn generates an inflammatory feedback loop. Disruption of this feedback loop should suppress inflammation and reestablish an appropriate immune response to the pathogen. Thus, the discovery of TLR inhibitors could result in the creation of potent treatment plans. Several researchers have suggested that TLR antagonists may be a potential way to control COVID‐19. The role of anti‐inflammatory factors in reducing death caused by persistent inflammation in the lungs of COVID‐19 patients has been shown. For example, CD24Fc couplers are used to block TLR activation.^5^ TLR4 antagonists have anti‐inflammatory effects on the lungs of mice with acute respiratory distress syndrome, protecting tissues from inflammation‐induced damage.^345^ However, some researchers hypothesize that using TLR antagonists improperly to treat COVID‐19 could lower IFN levels without inhibiting the virus. As a result, more research on TLR antagonist dosage and duration needs to be done at the clinical stage.^94^, ^346^

TLR antagonism has been successfully applied in various experimental models of cardiovascular disease(CVD). Animal experiments have shown that inhibiting TLR signaling can be used to treat or prevent atherosclerosis. Drugs that block TLR2‐ and TLR4‐dependent signaling pathways can lessen inflammatory activation pathways in atherosclerosis in mice and humans. Chloroquine, hydroxychloroquine, and quinacrine are three TLR‐related inhibitors that can be used to treat CVD because they prevent endosomal TLR activation and lower blood pressure and aortic endothelial dysfunction.^347^, ^348^ Kitazume et al.^349^ reported that ablation of the TLR9‐mediated signaling pathway attenuated myocardial ischemia/reperfusion injury and the inflammatory response.

## 7 CONCLUSION

Researchers first described TLR4 in 1997, and subsequently there has been a growing interest in the biological functions that TLRs serve in the body's immune system.^350^ TLRs activate two distinct signaling pathways—the canonical pathway via MyD88 protein and the noncanonical pathway through the TRIF. These proteins can also activate a variety of inflammatory cytokines or type I IFNs, which play an anti‐infection role. However, excessive activation of TLR signaling may cause pathological damage and even induce inflammatory and autoimmune diseases.

TLRs play an important role in inflammatory diseases and even in carcinogenesis. For example, they have been implicated in respiratory diseases associated with viral infections (COVID‐19), colitis associated with bacterial infections, and the pathogenesis of autoimmune diseases (T2DM). They have also been implicated in the pathogenesis of several human cancers, including CRC and BC. Since 2005, when researchers discovered that TLRs are expressed alongside tumor cells and help tumors evade immune surveillance, an increasing number of studies have focused on TLRs as new targets for cancer therapy.^351^

Although research related to TLR therapy has stalled for a while, following the approval of several checkpoint blockers for the treatment of melanoma patients, TLR agonists have re‐entered the picture as adjuvant agents for immunotherapy, and researchers hope to treat cancer by combining TLR agonists and immune checkpoint blockers.^328^, ^329^, ^330^, ^331^ Several scholars expected to eliminate the immunosuppression of DCs in the tumor environment by using different TLR agonists to activate DCs and subsequent antitumor immune responses. Recently, after the discovery of the role of TAMs in influencing tumor development, targeting TAMs with TLR agonists to alter polarity, eliminate phagocytic support of tumors and actively promote antitumor immune effects may be a direction for future research.^352^, ^353^, ^354^, ^355^, ^356^

However, TLRs still have many unexplored roles in very complex mammalian/human biological systems. Further in‐depth TLR‐related studies will improve our understanding of TLR signaling pathways, help to elucidate signaling pathways and disease mechanisms, and provide new targets and approaches for the development of therapies for a variety of infectious and autoimmune diseases and cancers.

## AUTHOR CONTRIBUTIONS

Y. L. conceived and designed the structure of this manuscript. K. W. and H. H., Q. Z., and H. D. wrote the paper. Y. L. revised the paper. All authors have read and approved the final manuscript.

## CONFLICT OF INTEREST STATEMENT

The authors declare that they have no conflict of interest.

## ETHICS STATEMENT

No ethical approval was required for this study.

## Data Availability

Not applicable.
